# Genome Sequence of a Dengue Virus Serotype 2 Strain Identified during the 2019 Outbreak in Bangladesh

**DOI:** 10.1128/MRA.01246-20

**Published:** 2021-01-07

**Authors:** Roly Malaker, Mohammad S. I. Sajib, Apurba R. Malaker, Hafizur Rahman, Yogesh Hooda, Md Hasanuzzaman, Samir K. Saha, Senjuti Saha

**Affiliations:** a Child Health Research Foundation, Dhaka, Bangladesh; b MRC Laboratory of Molecular Biology, Cambridge, United Kingdom; c Dhaka Shishu (Children) Hospital, Dhaka, Bangladesh; d Bangladesh Institute of Child Health, Dhaka, Bangladesh; Portland State University

## Abstract

A nearly complete genome sequence of a dengue virus serotype 2 strain detected in the serum of a patient in 2019 during the largest outbreak of dengue fever in Bangladesh is reported.

## ANNOUNCEMENT

Dengue is a mosquito-borne viral disease caused by dengue virus, a single-stranded positive-sense RNA virus of the family *Flaviviridae* (1). Here, we report the genome sequence of a dengue virus detected in a clinically symptomatic patient during the 2019 dengue outbreak in Bangladesh (2).

All protocols were approved by the ethical review board of the Bangladesh Institute of Child Health. Samples were collected for clinical care and diagnostic testing at the discretion of the attending physicians, and written consent was obtained from the caregiver for additional laboratory analysis.

For this study, a subset of dengue virus-positive serum specimens, tested using the NS1 antigen kit (product no. 09DEN10D; SD Biosensor, South Korea), were retested using reverse transcription-quantitative PCR (RT-qPCR) to estimate the dengue viral load (3). One specimen collected on 27 July 2019 in Dhaka, Bangladesh, had the highest viral load, with a cycle threshold value of 24, and was selected for next-generation sequencing. For library preparation, viral nucleic acid was extracted from a 200-μl serum sample using the Quick-DNA/RNA microprep extraction kit (product no. D7005; Zymo, CA, USA) according to the manufacturer’s protocol. The NEBNext Ultra II RNA library preparation kit (product no. E7770; New England Biolabs, MA, USA) was used to prepare the sequencing library. External RNA Controls Consortium Collection spike-in control mix (product no. 4456740; Thermo Fisher Scientific, MA, USA) was used as a marker of potential library preparation errors. The final library was sequenced on an Illumina iSeq100 sequencer using 150-nucleotide paired-end sequencing.

The library generated 7,688,000 reads, and 2,467,976 reads passed the default filter of BaseSpace (Illumina). Raw fastq files were uploaded to the IDseq portal to identify the pathogenic organisms, as described elsewhere (4, 5). In brief, the human reads were filtered out from the fastq file by alignment with a reference database using the Spliced Transcripts Alignment to a Reference (STAR) algorithm. Next, a series of quality control steps were performed to exclude low-quality reads, duplicates, and low-complexity reads with the help of the Paired-Read Iterative Contig Extension (PRICE) package, the CD-HIT-DUP tool (v4.6.8), and the Lempel-Ziv-Welch (LZW) algorithm. Another round of host filtering was executed by applying the Bowtie2 package. The remaining reads were queried against the most recent version of the NCBI nucleotide and nonredundant protein databases using Genomic Short-read Nucleotide Alignment Program (GSNAP) (v2018-10-26) and RAPSearch 2 (v2.24), respectively. Finally, the matched reads and their relative abundance were calculated and mapped at the genus level and visualized.

**FIG 1:**
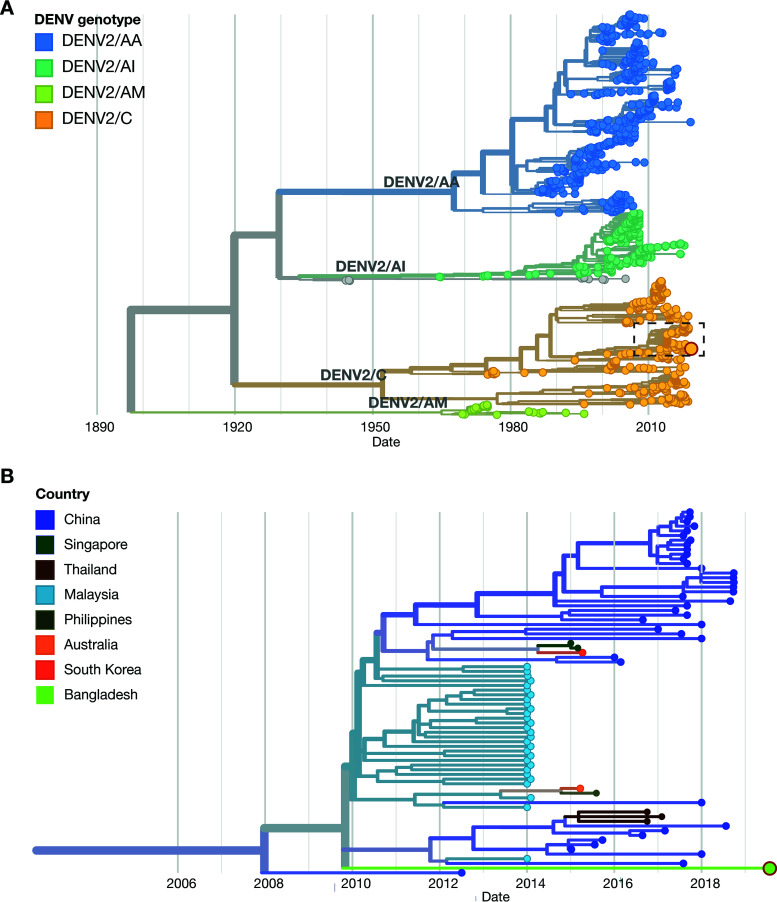
Phylogenetic trees of dengue virus serotype 2 (DENV2) strains rendered using Nextstrain. (A) Phylogenetic tree of CHRF_DenV002 (GenBank accession no. MN328061) contextualized with 1,202 dengue virus serotype 2 genomes available on Nextstrain. The circle bordered in red represents the position of CHRF_DenV002 in the tree. (B) Phylogenetic tree of CHRF_DenV002 contextualized with the closest genomes sequenced from Southeast and East Asian countries (access date, 18 October 2020).

The final nearly complete genome of the dengue virus consisted of 10,699 bases, with a GC content of 45.8% and an average coverage depth of 237×. It had 92.5% nucleotide and 97.5% amino acid sequence identity to the reference dengue virus serotype 2 genome (GenBank accession no. NC_001474.2). The genome was submitted to the NCBI GenBank database (accession no. MN328061) and subsequently contextualized in the dengue virus serotype 2 build of Nextstrain (https://nextstrain.org/dengue/denv2) among 1,202 available dengue virus serotype 2 genomes sampled between 1944 and 2019 (6) (Fig. 1A). The sequenced genome is closely related to dengue viruses seen in other Southeast and East Asian countries, specifically, Singapore, Malaysia, Indonesia, South Korea, and China (Fig. 1B).

### Data availability.

The dengue viral genome from Bangladesh has been deposited in GenBank (accession no. MN328061). The raw reads have been deposited in the NCBI Sequence Read Archive (SRA accession no. SRR12901070). The BioProject and BioSample accession numbers are PRJNA672124 and SAMN16557024, respectively.
